# Metabolic alterations in the nymphal instars of *Diaphorina citri* induced by *Candidatus* Liberibacter asiaticus, the putative pathogen of huanglongbing

**DOI:** 10.1371/journal.pone.0191871

**Published:** 2018-01-25

**Authors:** Nabil Killiny, Shelley E. Jones

**Affiliations:** Citrus Research and Education Center, Department of Plant Pathology, IFAS, University of Florida, Lake Alfred, Florida, United States of America; Agricultural Research Organization Volcani Center, ISRAEL

## Abstract

Currently, huanglongbing is the most damaging disease of citrus causing huge economic losses. The disease is caused by the Gram-negative bacterium *Candidatus* Liberibacter asiaticus (*C*Las). The pathogen is transmitted in a persistent propagative circulative manner within its vector, the Asian citrus psyllid, *Diaphorina citri*. Exploring the metabolic alteration in the vector may lead to a better understanding of the nutritional needs of *C*Las and to designing an artificial medium for culturing the pathogen. It has been shown that the nymphal stages have a greater role in transmission mainly because they feed on plants more actively than adults. In this study, we carried out an untargeted comparative metabolomic analysis for healthy and *C*Las-infected 4^th^ / 5^th^ instar nymphs. The metabolic analysis was performed using trimethylsilylation and methyl chloroformate derivatization followed by Gas Chromatography-Mass Spectrometry (GC-MS). Overall, the changes in the nymph metabolism due to the infection with *C*Las were more pronounced than in adults, as we previously published. Nymphs reared on *C*Las-infected Valencia sweet orange were higher in many metabolites, mainly those of the TCA cycle, C16 and C18 fatty acids, glucose, sucrose, L-proline, L-serine, pyroglutamic acid, saccharic acid, threonic acid and *myo*-inositol than those reared on healthy plants. In contrast, *C*Las-infected nymphs were lower in putrescine, glycine, L -phenylalanine, L -tyrosine, L -valine, and *chiro*-inositol. The information provided from this study may contribute in acceleration of the availability of *C*Las in culture and consequent screening of antibacterial compounds to discover a definitive solution for huanglongbing.

## Introduction

The Asian citrus psyllid, *Diaphorina citri* Kuwayama (Hemiptera: Liviidae) has been under intense scrutiny since the arrival of citrus Huanglongbing (HLB) disease to the citrus producing areas of the world. This destructive disease is spread from tree to tree by *D*. *citri* during feeding activities. The disease itself is thought to be the result of a plant pathogen, *Candidatus* Liberibacter asiaticus (*C*Las), although to date, it has not been cultured and Koch’s postulate has not been proven. However, the fastidious bacterium has been found in plants showing symptoms of HLB, as well as from local psyllid populations, and its genome has been sequenced. *C*Las is a phloem-restricted α-Proteobacteria containing one circular chromosome with a GC ratio of about 36% [1]. Psyllids feed heavily on the phloem of citrus and other species of the Rutaceae family, including ornamentals such as curry tree (*Bergera koenegii*) and orange jasmine (*Murraya paniculata*). *D*. *citri* nymphs arise from eggs laid on the soft, new flush of citrus trees several times a year.

Citrus greening disease, or HLB, was first linked to presence of the Asian citrus psyllid in Florida in 2004 [2], but has been a well-described disease of citrus for many years [3]. HLB causes a variety of foliar symptoms, small and bitter fruit that are unfit for processing, and eventual tree death for most citrus varieties. It has decimated citrus production in the geographical areas where it is present, especially in Florida, where it has affected nearly 100% of citrus groves [4]. Further complicating the control of HLB in citrus groves are the lack of foliar symptoms in early stages of infection, uneven distribution of bacteria within trees (leading to false negative diagnoses), and difficulty in culturing the bacteria which would allow development of pathogen-specific antibacterial treatments.

Although adult *D*. *citri* can acquire *C*Las while feeding from infected citrus, *C*Las acquisition is most efficient during the nymph stage [5,6] and, therefore, may have a bigger impact on the nymph metabolism. After first acquisition of the bacteria, *C*Las titer was higher within nymphs compared to adults, and more than 50% of nymphs tested positive for *C*Las compared to less than 30% acquisition rate for adult psyllids [6]. Transmission rates from *C*Las-infected *D*. *citri* nymphs to citrus seedlings were found to be about 20% compared to 0% from infected adults [6]. Growing nymphs feed on phloem at a much higher rate than adults, producing large quantities of honeydew compared to adults. More intensive feeding behavior may give a greater opportunity for nymphs to acquire *C*Las from infected host plants, and then become highly inoculative adults which are more likely to transmit the disease to healthy citrus trees than psyllids that acquired *C*Las as adults. Furthermore, the expression of most of nymph proteins involved in bacterial adhesion and immunity were found to be less responsive to bacterial invasion than that of adult psyllids exposed to *C*Las, suggesting that the innate immune system of *D*. *citri* nymphs is reduced to encourage colonization by bacterial symbionts [5].

Many attempts to culture Liberibacter *spp*. which include *Ca*. Liberibacter asiaticus in the U.S. and Asia, and *Ca*. Liberibacter americanus in Brazil [7], have met with some degree of success such as some successive passages in enriched media [8–11], but the cultures have not been sustainable. Only *Liberibacter crescens*, a relative infecting the mountain papaya, has been successfully cultured *in vitro* [12,13]. Genomic comparisons of *C*Las with cultivable *L*. *crescens* revealed a lack of genes related to production of fatty acids and aromatic amino acids (phenylalanine, tryptophan, tyrosine). This data suggests that *C*Las must rely on either the host plant or insect vector for these essential nutrients and, therefore, they should be included in the development of any potential growth medium for *C*Las.

Recently, we studied the metabolite contents of adult *D*. *citri* and found that metabolite levels were significantly higher in *C*Las-infected adult psyllids than in non-infected psyllids, especially those involved in the tricarboxylic acid (TCA) cycle [14]. Genes controlling enzymes involved in citric acid metabolism, including succinate dehydrogenase, 2-oxoglutarate dehydrogenase-like, and succinate-semialdehyde dehydrogenase, were found to be upregulated up to 2.6 times in *C*Las-infected *D*. *citri* compared to control adult psyllids [6]. In this follow-up study, we focused on identifying the polar metabolites of *D*. *citri* nymphs reared on ‘Valencia’ sweet orange trees considered “healthy” (*C*Las-negative by PCR) or “*C*Las-infected” (*C*Las-positive by PCR) and hypothesized that alterations of the TCA cycle metabolism will be more pronounced in nymphs reared on *C*Las-infected trees, than those reared on healthy Valencia trees. We expect these changes to reveal specific clues to the nutrient requirements for *C*Las, enabling us to better define a medium for axenic culture.

## Materials and methods

### Asian citrus psyllid (*Diaphorina citri*) cultures

*D*. *citri* colonies were reared on healthy or *C*Las-infected *Citrus sinensis* 'Valencia' sweet orange nursery trees which previously tested negative or *C*Las-positive by PCR. Mesh cages containing the psyllid colonies were kept in temperature controlled growth chambers (27±2 °C, 70±5% RH, 14:10 h photoperiod) located at the CREC, Lake Alfred, FL. Nymphs were transferred from citrus branches to a damp filter paper using a soft #2 camel-hair brush, and sets of 100 nymphs of 4^th^ and 5^th^ instar were then individually selected for the study.

### Feeding assay

*D*. *citri* adults or 4^th^/5^th^ instar nymphs were placed into 0.25 mL microcentrifuge tubes with a disc of filter paper (made with a hole puncher). Each disc was dampened with 10 μL of 20% sucrose for feeding. Three adults or three nymphs were placed in each of five prepared tubes and left for 18h in the growth room at 27 °C. Two blank samples per treatment were made with paper disks treated with 20% sucrose, but without psyllids. At the end of the feeding period, the nymphs or adults were carefully collected from the tubes. The filter papers were allowed to dry under the fume hood for 2 h before the ninhydrin test. For the test, a solution of 1% ninhydrin in acetone was prepared fresh (1 g/100 mL acetone). Each disc was dipped into the solution for 3 min, transferred to a paper towel for drying. Color was allowed to develop at room temperature for 1 h. Discs with more color development were interpreted as having had more feeding activity or more visits to the sucrose disc by the nymphs or adult psyllids.

### Nymph metabolite extraction

Further collections of *D*. *citri* nymphs (in sets of 100 nymphs) were made for metabolite extraction. Each set of 100 nymphs was placed into a pre-weighed screw cap centrifuge tube (02-707-354, Fisher Scientific) and re-weighed to determine the nymph mass. Five sets each of 100 nymphs from healthy and *C*Las-infected citrus flush were prepared. Tubes were then flash frozen in liquid nitrogen for 5 min or until boiling stopped. A 5 mm stainless steel ball was added to each tube for homogenization using a Tissuelyser II (85300, Qiagen). Into each tube of nymph powder, 500 μL of extraction solvent (8:1:1 methanol:water:chloroform) was added and the nymph homogenates were extracted overnight on a rotating mixer at 6 °C. Following extraction, the tubes were centrifuged for 5 min at 10,000 rpm to remove debris. Supernatants were transferred to clean 1.5 mL microcentrifuge tubes, and two aliquots of 100 μL of each extract were taken for GC-MS analysis. One portion was chemically derivatized using trimethylsilylation (TMS) protocol [14,15], while the second portion was reserved for derivatization using the methyl chloroformate (MCF) protocol [16,17].

### Derivatization for GC-MS analysis

For detection of a broad range of metabolites, including amino acids, organic acids and sugar compounds, each methanolic *D*. *citri* nymph extract (100 μL) was spiked with 10 μL of ribitol (0.5 mg/mL, A5502, Sigma-Aldrich) as internal standard, and dried under a stream of nitrogen. Dried nymph residues were silylated as described previously [14,15]; GC-MS column and conditions were identical to those reported therein. There were five biological replicates each of healthy and infected nymph extracts which were injected in duplicate (n = 10).

For MCF derivatization, heptadecanoic acid (0.5 mg/mL, H3500, Sigma-Aldrich) was added as internal standard, (10 μL) and alkylation was performed as described by Killiny and Nehela [16,17] with modifications. To each sample (100 μL), 450 μL of 1N NaOH, 425 μL cold methanol (A452, Fisher Scientific), and 88 μL pyridine (364421000, Acros) were added, with vortexing after each addition. Methyl chloroformate (430841000, Acros) was added in two additions of 50 μL followed by 30 s vortexing after each addition. Chloroform (2683200, Acros) was added (125 μL), with vortexing for 30 s. Finally 500 μL of 50 mM NaHCO_3_ was added with a final vortex of 30 s. From the lower organic phase, 75 μL was transferred into an amber glass GC vial with 300 μL insert (C4000-LV2, National Scientific) containing ~ 1 mg of sodium sulfate crystals for drying; 0.5 μL was injected into the GC-MS splitlessly. Each sample was injected twice (n = 10 injections each for healthy, n = 10 for infected). Two blank samples containing only the derivatized internal standard were injected and artifact peaks were removed from the data analysis.

### Metabolite identification

Metabolites scoring at least 700/1000 in library matching hits using NIST 2011 (National Institutes of Standards and Technology, Gaithersburg, MD) and Wiley 9^th^ ed. Mass Spectral Library (Wiley & Sons, Hoboken, NJ) were further identified by a combination of parameters: 1) by comparison with mass spectra and linear retention index (RI) values previously confirmed in our laboratory using authentic reference standards treated identically to samples; 2) with that of values found in published literature and/or the Golm Metabolome Database (http://gmd.mpimp-golm.mpg.de/). Metabolites present consistently in samples but not found in a commercial or in-house database were designated as “unknown” and listed with their major *m/z* ion fragments.

### Statistical analysis

Data was normalized by both the mean peak area of the internal standard and the mean mass of the nymph samples. On column mass was calculated to be 10–30 μg per injection. Two-tailed, paired *t*-tests were performed to compare between the compound amounts in healthy and *C*Las-infected nymphs. Comparisons were considered significantly different if the *p*-value was less than 0.05. Fold changes were calculated by dividing the mean normalized peak areas of infected nymph samples by that of the healthy nymphs.

## Results and discussion

### *D*. *citri* nymphs feed more actively than adults

**Fig 1:**
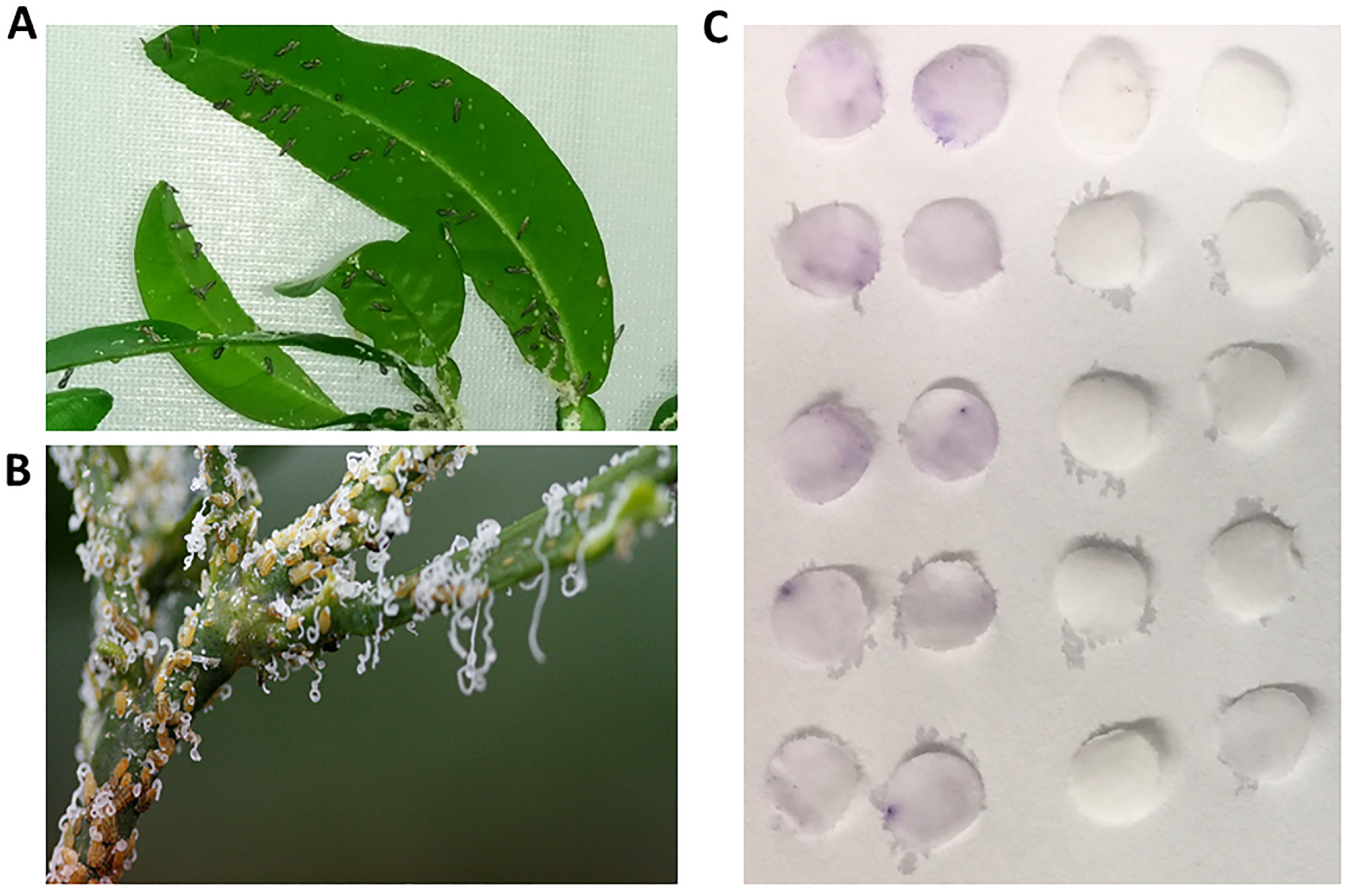
Feeding activity of nymphs and adults of *Diaphorina citri*. **A**: Adult Asian citrus psyllids (*Diaphorina citri*) feeding on phloem sap from citrus stems and leaves. Note droplets of honeydew on undersides of leaves. **B**: *D*. *citri* nymphs produce large quantities of waxy secretions when feeding, much more compared to adults. **C**: Feeding assay with ninyhydrin after feeding psyllids on sucrose-loaded discs of filter paper. Discs were stained with the amino acid specific dye, ninhydrin. The first two columns (left) are for nymphs, followed by two columns (right) for adults.

The feeding habits of adults and nymphal psyllids are quite different, as shown in Fig 1. Nymphs ingest large amounts of phloem sap and excrete the excess in the form of waxy secretions while adults eliminate excess sugar and waste as clear droplets or white pellets. Additionally, measurement of feeding event by Electrical Penetration Graph (EPG) was recently carried out on adult and nymph *D*. *citri*, and found the mean phloem feeding ingestion event time for nymphs was 5.3 h vs. 1.3 h for adults [18]. Subsequently, we developed an alternative assay using a paper disc saturated with 20% sucrose to evaluate feeding activity. The results of the feeding assay showed that nymphs visited the sucrose discs more often than adult psyllids as was shown by a larger number of amino acid spots darkened by ninhydrin (Fig 1C).

### Infection with *C*Las does not alter the mass of *D*. *citri* nymphs

The mean fresh weight of 100 *D*. *citri* 4^th^-5^th^ instar nymphs was 28.1±3.0 mg and 23.0 ±9.6 mg reared on healthy or *C*Las-infected ‘Valencia’ sweet orange trees respectively. An Excel *t*-test showed the values were not significantly different (*p* = 0.26).

### Summary of GC-MS analyses

In total, we detected 51 nymph metabolites by TMS derivatization and GC-MS, and 27 metabolites by MCF derivatization and GC-MS, with some overlap of compounds in common. By chemical class, there were five non-proteinogenic amino acids (NPAA), 10 amino acids (AA), five organic acids (OA), three sugar acids (SA), 11 sugars, eight sugar alcohols, three fatty acids (FA), and three sugar phosphates, phosphoric acid, an indole-like compound, and one unknown using TMS. By MCF there were three NPAA, 14 AA, five OA, one SA, and four FA. By mass (ng) percent, sugars represented 78.3% and 82.5% of the metabolite mass in healthy and *C*Las-infected nymphs, respectively, followed by sugar alcohols (8.6%, 5.3%), OAs (5%, 6%) and about 5% of AA and NPAA combined for both sets of samples. For percentage calculations, the TMS results were used because it more accurately represents the broad range of polar metabolites that may be present in nymphs, compared to MCF which preferentially derivatizes amines and carboxylic groups. In addition, we found important differences between individual metabolites of each chemical class from extracts of nymphs reared on healthy or HLB+ Valencia trees.

### Proteinogenic and non-proteinogenic amino acids

Among the 24 different amines detected by GC-MS in healthy and infected nymphs of *D*. *citri*, six were found to be significantly different. Amino acids which were increased by in response to *C*Las by either GC method included L-proline (*p* = 0.00001), L-serine (*p* = 0.002) and pyroglutamic acid (*p* = 0.023) as shown in Tables 1 and 2. Those which decreased were putrescine, L-glutamic acid, L-tryptophan, L-tyrosine, L-phenylalanine and L-valine. L-Proline increased in response to *C*Las by 2.3-fold as detected by TMS derivatization, and 1.6-fold by MCF (Tables 1 and 2). Elevation of proline is considered a general biotic stress response which has also been observed in *C*Las-infected citrus plants [17]. Interestingly, in our previous study of the effects of *C*Las infection on adult *D*. *citri* [14], proline was reduced to nearly half the control level. However, in adults, glycine, L-serine and L-threonine increased. Likewise, in *D*. *citri* nymphs, the trend was similar for serine and threonine, but glycine decreased significantly, suggesting some utilization of glycine by *C*Las, or that the glycine decrease was related to the significant accumulation of serine and threonine (Table 2). Glycine and serine share a reversible metabolic pathway regulated by glycine hydroxymethyltransferase [EC:2.1.2.1] [19]. Serine and threonine play a role in plant defense response, and were found to be higher in Valencia leaves exposed to *C*Las and *D*. *citri* [17]. Furthermore, glycine levels were of relatively low abundance in nymphs compared to adults, and was not detected at all by MCF in nymph samples.

**Table 1:** Concentration (ng∙nymph^-1^) of TMS-derivatized polar metabolites of *D*. *citri* nymphs reared on healthy or *C*Las-infected Valencia sweet orange flushes.

^a^Metabolite name/*class*	Healthy (Mean ± SD)			*C*LAS-infected (Mean ± SD)			*P* value	^b^Fold change
2-Aminopropanol	0.08	±	0.01	0.10	±	0.04	0.348	1.23
β-Alanine^a^	0.05	±	0.02	0.05	±	0.04	0.720	-1.13
γ-Aminobutyric acid (GABA) ^a^	0.30	±	0.06	0.48	±	0.24	0.094	1.62
Putrescine^a^	0.59	±	0.15	0.29	±	0.23	**0.023**	-**2.04**
Pyroglutamic acid^a^	0.63	±	0.28	1.74	±	0.98	**0.023**	**2.76**
***Total NP Amino Acids***	*1*.*66*	±	*0*.*51*	*2*.*66*	±	*1*.*53*		*1*.*61*
L-Alanine^c^	1.65	±	0.65	1.39	±	0.70	0.522	-1.19
L-Aspartic acid^c^	0.23	±	0.43	0.07	±	0.08	0.408	-3.13
L-Glutamic acid^c^	0.35	±	0.37	1.58	±	1.69	0.112	4.56
Glycine^c^	1.42	±	0.48	0.87	±	0.30	**0.041**	-**1.62**
L-Isoleucine^c^	0.14	±	0.04	0.15	±	0.09	0.823	1.07
L-Phenylalanine^c^	0.02	±	0.01	0.12	±	0.13	0.071	7.66
L-Proline^c^	1.03	±	0.61	2.36	±	1.89	0.131	2.30
L-Serine^c^	0.12	±	0.05	0.14	±	0.11	0.755	1.13
L-Threonine^c^	0.12	±	0.11	0.20	±	0.10	0.166	1.76
L-Valine^c^	0.49	±	0.23	0.29	±	0.19	0.134	-1.66
***Total Amino acids***	*5*.*55*	±	*2*.*97*	*7*.*19*	±	*5*.*28*		*1*.*29*
Citric acid^c^	3.23	±	2.09	7.25	±	2.44	**0.012**	**2.78**
Malic acid^c^	1.43	±	0.92	2.92	±	1.05	**0.026**	**2.04**
Quinic acid^c^	2.08	±	1.04	2.64	±	0.97	0.355	1.51
Succinic acid^c^	0.18	±	0.05	0.25	±	0.16	0.336	1.40
Threonic acid^c^	0.01	±	0.01	0.03	±	0.02	**0.005**	**4.21**
***Total Organic acids***	*6*.*92*	±	*4*.*11*	*13*.*07*	±	*4*.*65*		*1*.*89*
2-Ketogluconic acid^c^	*Trace*			*Trace*			**0.007**	**3.69**
Gluconic acid^c^	0.02	±	0.01	0.09	±	0.08	**0.042**	**4.32**
Saccharic acid^c^	*Trace*			0.01		0.00	**0.001**	**11.96**
***Total Sugar acids***	*0*.*02*	±	*0*.*01*	*0*.*10*	±	*0*.*08*		*4*.*66*
Arabinopyranose^a^	0.10	±	0.10	0.09	±	0.08	0.725	-1.18
Fructose^c^	6.33	±	3.11	8.75	±	2.89	0.193	1.38
Glucofuranoside, methyl^a^	0.38	±	0.37	0.44	±	0.32	0.761	1.16
Glucopyranose^a^	0.50	±	0.79	0.62	±	0.85	0.801	1.24
Glucose^c^	54.31	±	22.22	79.26	±	27.03	0.111	1.46
Lyxose^c^	0.44	±	0.25	0.25	±	0.06	**0.003**	-**2.14**
Sucrose^c^	29.01	±	8.41	76.63	±	26.50	**0.002**	**2.73**
Trehalose^c^	15.90	±	24.29	14.44	±	11.08	0.897	-1.11
Unk *m/z* 217/437	0.05	±	0.04	0.08	±	0.05	0.225	1.67
Unk *m/z* 217/302	0.29	±	0.03	0.26	±	0.13	0.590	-1.11
Xylose^c^	0.02	±	0.01	0.04	±	0.03	0.050	2.41
***Total Sugars***	*107*.*33*	±	*59*.*62*	*180*.*87*	±	*69*.*03*		*1*.*69*
Glycerol^c^	0.47	±	0.12	0.75	±	0.35	0.085	1.62
Erythritol^c^	0.03	±	0.01	0.03	±	0.01	0.929	-1.02
Xylitol^c^	0.05	±	0.03	0.07	±	0.03	0.405	1.29
Glucitol^c^	2.07	±	1.13	2.43	±	1.34	0.630	1.17
*chiro*-Inositol^c^	0.65	±	0.51	0.05	±	0.03	**0.016**	-**12.51**
*scyllo*-Inositol^c^	0.14	±	0.04	0.28	±	0.15	**0.045**	**2.04**
*myo*-Inositol^c^	8.37	±	0.71	7.93	±	2.94	0.731	-1.06
Unk Sugar alcohol *m/z* 217/318	0.07	±	0.05	0.07	±	0.03	0.880	-1.03
***Total Sugar alcohols***	*11*.*79*	±	*2*.*55*	*11*.*55*	±	*4*.*85*		*-1*.*02*
Palmitoleic acid^c^	0.02	±	0.01	0.03	±	0.02	0.308	1.44
Palmitic acid^c^	0.03	±	0.02	0.05	±	0.05	0.273	1.92
Stearic acid^c^	0.03	±	0.02	0.06	±	0.02	**0.013**	**2.18**
***Total Fatty acids***	*0*.*07*	±	*0*.*05*	*0*.*14*	±	*0*.*08*		*1*.*89*
α-Glycerophosphate^c^	0.15	±	0.05	0.11	±	0.07	0.247	-1.38
Inositol-2-Phosphate^a^	0.01	±	0.01	0.01	±	0.00	0.794	1.13
*myo*-Inositol-5-phosphate^a^	0.01	±	0.01	0.07	±	0.03	**0.001**	**5.85**
Phosphoric acid^c^	3.43	±	1.12	3.42	±	1.32	0.994	1.00
***Total Phospho-compounds***	*3*.*61*	±	*1*.*18*	*3*.*62*	±	*1*.*43*		*1*.*00*
Unk *m/z* 263	0.03	±	0.02	0.05	±	0.06	0.348	1.89
1H-Indole-3-glycerophosphate^a^	0.04	±	0.02	0.05	±	0.03	0.156	1.55
***Other***	*0*.*06*	±	*0*.*03*	*0*.*11*	±	*0*.*09*		*1*.*70*
***Total ng/nymph***	***137*.*01***	±	***71*.*03***	***219*.*31***	±	***87*.*01***		***1*.*60***

Five replicates of ~100 nymphs were extracted, with duplicate injections (*n* = 10) into the GC-MS.

^a^Mass spectra matching entries in NIST 2011 and Wiley 9th ed. with a score of >700/1000 were tentatively identified and compared with published RI values.

^b^Fold changes and Excel *t*-tests were performed using the peak areas normalized by both insect mass and the mean response of the internal standard. Entries marked *Trace* were below the limit of quantification but generated a small peak area.

^c^Compounds were confirmed using derivatized reference substances.

**Table 2:** Concentration (ng∙nymph^-1^) of MCF-derivatized polar metabolites of *D*. *citri* nymphs reared on healthy or *C*Las-infected Valencia sweet orange flushes.

^a^Metabolite name/*class*	Healthy Mean ± SD			Infected (Mean ± SD)			*P* value	^b^Fold change
γ-Aminobutyric acid (GABA)^c^	0.044	±	0.013	0.043	±	0.010	0.872	-1.01
Pyroglutamic acid^c^	*Trace*			*Trace*			0.546	1.11
Tyramine^c^	0.008	±	0.002	0.007	±	0.002	0.572	-1.08
***Total NP Amino Acids***	***0*.*05***	±	***0*.*02***	***0*.*05***	±	***0*.*01***		***-1*.*03***
L-Alanine^c^	0.295	±	0.046	0.289	±	0.046	0.775	-1.02
L-Cysteine^c^	0.006	±	0.004	0.006	±	0.002	0.648	-1.11
L-Glutamine^c^	0.130	±	0.061	0.094	±	0.137	0.483	-1.19
L-Histidine^c^	*Trace*			*Trace*			0.346	-1.43
L-Leucine^c^	0.077	±	0.013	0.065	±	0.014	0.063	-1.20
L-Lysine^c^	*Trace*			*Trace*			0.199	-1.32
L-Methionine^c^	0.023	±	0.005	0.034	±	0.023	0.189	1.46
L-Phenylalanine^c^	0.030	±	0.005	0.018	±	0.014	**0.025**	-**1.68**
L-Proline^c^	0.228	±	0.041	0.413	±	0.078	**0.000**	**1.57**
L-Serine^c^	0.011	±	0.006	0.025	±	0.010	**0.002**	**2.33**
L-Threonine^c^	0.003	±	0.001	0.004	±	0.003	0.525	1.25
L-Tryptophan^c^	*Trace*			*Trace*			0.473	1.25
L-Tyrosine^c^	0.099	±	0.013	0.067	±	0.022	**0.002**	-**1.47**
L-Valine^c^	0.347	±	0.074	0.268	±	0.035	**0.010**	-**1.27**
***Total Amino Acids***	***1*.*25***	±	***0*.*27***	***1*.*28***	±	***0*.*38***		***1*.*03***
2-ketoglutaric acid^c^	0.21	±	0.06	0.17	±	0.06	0.094	-1.31
Anisic acid^a^	0.25	±	0.04	0.11	±	0.04	**0.000**	-**2.90**
Citric acid^c^	0.06	±	0.02	0.11	±	0.05	**0.024**	**2.45**
Malic acid^c^	0.90	±	0.32	1.15	±	0.22	0.071	1.28
Salicylic acid^c^	0.30	±	0.10	0.12	±	0.04	**0.000**	-2.68
Succinic acid^c^	0.72	±	0.19	0.83	±	0.16	0.170	1.17
***Total Organic acids***	***2*.*44***	**±**	***0*.*68***	***2*.*31***	**±**	***0*.*51***		***-1*.*05***
Palmitoleic acid^c^ (C16:1)	25.10	±	3.12	30.26	±	3.67	**0.005**	**1.21**
Palmitic acid^c^	3.89	±	0.52	4.59	±	0.66	**0.024**	**1.18**
Linoleic acid^c^ (C18:2)	26.64	±	4.65	39.64	±	14.22	**0.019**	**1.49**
Stearic acid^c^ (C18:0)	6.46	±	1.90	8.97	±	1.41	**0.006**	**1.39**
Oleic acid^c^ (C18:1)	54.27	±	7.32	46.77	±	30.72	0.487	-1.16
Arachidic acid^a^ (C20:0)	3.10	±	1.67	4.66	±	1.69	0.067	1.51
***Total Fatty acids***	***119*.*46***	±	***19*.*18***	***134*.*88***	±	***52*.*36***		***1*.*13***
***Total ng/nymph***	***123*.*20***	±	***20*.*14***	***138*.*53***	±	***53*.*27***		***1*.*12***

Five replicates of ~100 nymphs were extracted, with duplicate injections (*n* = 10) into the GC-MS.

^a^Mass spectra matching entries in NIST 2011 and Wiley 9th ed. with a score of >700/1000 were tentatively identified and compared with published RI values.

^b^Fold changes and Excel *t*-tests were performed using the peak areas normalized by both insect mass and the mean response of the internal standard. Entries marked *Trace* were below the limit of quantification but generated a small peak area.

^c^Compounds were confirmed using derivatized reference substances.

The concentration of pyroglutamic acid (PGA), the most abundant NPAA found in adult *D*. *citri* and haemolymph of *D*. *citri* [14,20] was higher in *C*Las-infected nymphs than in healthy nymphs (*p* = 0.023) in this study. These results are in opposition to our study of adult metabolites, where levels were lower in infected adult psyllids. It was thought that the increase of γ-aminobutyric acid (GABA) from its PGA precursor resulted in lower levels of PGA. In the present study, an increase in PGA may indicate an inhibition of GABA biosynthesis in nymphs due to *C*Las or a not yet understood role for PGA in developing nymphs.

Tyrosine was significantly reduced in response to *C*Las (*p* = 0.002). Tyrosine is critical in the synthesis of cuticular proteins of the exoskeleton of aphids [21] and other insects. In the pea aphid, *Acyrthosiphon pisum*, tyrosine is synthesized by one of its endosymbiont bacteria, *Buchnera aphidicola* [21]. Nutritional symbionts are common in most insects, especially hemipterans, whose specialized diets are nutrient poor and who may not obtain all of the amino acids from the sugar rich phloem sap upon which they feed exclusively [5,22]. However, tyrosine is readily available from citrus phloem sap. Previously, we detected tyrosine in healthy Valencia phloem sap by GC-MS [23,24]. It may be that HLB-stressed trees have lower amounts of available tyrosine or that *C*Las utilizes the tyrosine ingested by feeding nymphs, since the *C*Las genome predicts that it can metabolize tyrosine but cannot produce it [1].

Valine was significantly lower in *C*Las infected nymphs (*p* = 0.01, Table 2). Proteins involved in valine catabolism were reported to be upregulated in adult *C*Las-infected psyllids by about 4-fold [25]. As a class of compounds, there was a net fold change of 1.29 and 1.03 by TMS and MCF, respectively for AAs; for the NPAA class, the net fold change was 1.61 for TMS and was down slightly (-1.03) for MCF-derivatized samples. The two methods did not detect all of the same amines, and so it is not surprising there was a lack of agreement in the results by metabolite class.

### Organic acids

The major organic acids detected in *D*. *citri* nymph extracts included citric, quinic and malic acids. In adult psyllids, we found that malic acid decreased while citric acid increased after *C*Las infection [14] indicating that the equilibrium of the TCA cycle was affected, perhaps by increased activity of malate dehydrogenase. In the present study of the nymph metabolome, citric acid increased more than two-fold in response to *C*Las, suggesting that infected nymphs may feed more heavily than healthy nymphs, or again, that *C*Las infection upregulates components of the citric acid cycle. The excellent work by Ramsey et al. [25] examining the differential expression of proteins found in *D*. *citri* and its endosymbionts gave us many clues to the metabolism changes induced by *C*Las. It was found that in psyllids harboring *C*Las along with their bacterial symbionts, proteins involved in the citric acid cycle were upregulated. In *Proftella armatura*, one of the several endosymbionts of *D*. *citri*, about 80% (15 of 18) of differentially expressed proteins were upregulated, including malate dehydrogenase, succinate dehydrogenase, and succinyl CoA synthetase subunit A [25]. In the same study, four proteins were differentially expressed (upregulated) significantly between *C*Las-infected and healthy psyllids, including those that produce intermediates succinate and 2-oxoglutarate in the citric acid cycle [25]. Therefore, it is probable that *C*Las manipulates not only *D*. *citri* metabolism but also that of its endosymbionts.

Several phenolic compounds were detected in *D*. *citri* nymph extracts, which were most likely of plant origin, acquired during feeding. Quinic acid was found to be ~ 30 mM in Valencia phloem sap [26], the food source for nymph and adult *D*. *citri* and was also detected in nymph extracts. The level was higher in nymphs reared on HLB+ Valencia, which fits with previous findings of elevated quinic acid in infected Valencia, probably as a plant defense against *C*Las [27]. Salicylic acid (2-hydroxybenzoic acid) and *o*-anisic acid (2-methoxybenzoic acid) were both significantly lower (almost 3-fold) in response to *C*Las. Also of plant origin, the presence of these compounds in nymphs is most likely due to feeding or direct contact with the plant surfaces. Valencia sweet orange is one of the most HLB-susceptible varieties of citrus, and previous studies showed an increase in SA-related compounds, most likely due to activation of SA-mediated plant defenses [17,28]. Lower SA content in nymphs with *C*Las could be due to translocation of SA out of the phloem sieve tubes and into the leaves where plant defense is most needed [29]. Recently, Li et. al [30] found that the *C*Las genome encodes a SA-hydroxylase which may be able to degrade SA thereby suppressing the plant host response. This effect would support our results of the lower levels of SA found in nymph extracts fed on *C*Las+ Valencia trees.

### Mono and disaccharides

Fructose, glucose, sucrose and trehalose were the major carbohydrate metabolites present in nymph extracts, totaling 105 ng and 179 ng for nymphs reared on healthy and HLB+ Valencia respectively (Table 1). Of these, only sucrose was significantly different (*p* = 0.002) with a 2.7-fold increase in response to *C*Las. Higher levels of sucrose could be due to increased feeding by infected nymphs or to reduced invertase activity which converts sucrose to glucose and fructose in the alimentary canal. We suspect that invertase activity may be directly or indirectly suppressed by the *C*Las bacteria.

Trehalose is considered the blood sugar of insects [31], but we found glucose to be the most dominant sugar in the haemolymph [20] and whole adult *D*. *citri* [14]. We found a wide range of trehalose levels in nymph samples, which resulted in large standard deviations and a lack of significant differences. The values per nymph ranged from 5 to 65 ng for healthy nymphs and 5 to 36 ng in infected nymphs. Trehalose is a dimer of glucose which is formed in the gut of insects to reduce osmotic pressure and is the first source of carbohydrate energy utilized for flight [31]. When trehalose levels in the hemolymph reach optimum levels, the excess is shunted to the fat body for storage as triglyceride [32].

Monosaccharides such as fructose and glucose are critical for bacterial biofilm formation and virulence. Biofilm formation is essential to the pathogenicity of many circulative insect-vectored plant pathogens including *Xylella fastidiosa* (*Xf*), the cause of Pierce’s disease in grapes [33,34]. In the *Xf* model, exopolysaccharides (rather than lipopolysaccharides) are secreted as biofilms for attachment to the mouthparts of the blue-green sharpshooter, another hemipteran. The bacteria must acquire monosaccharides from the host or vector to grow and form polysaccharides for adhesion to the host’s structures until it is eventually released to circulate and exit the vector. Likewise, in order to colonize and grow in artificial media, the correct sugars must be supplied. Vector-to-plant transmissions of *Xf* were very low in mutant *Xf* missing the *gumD* and *gumH* genes (homologous to xanthan gum of *Xanthomonas campestris*). When *gum*-deficient *Xf* mutants were injected into grapevines they did not multiply and did not cause symptoms in plants [34]. Similarly, *X*. *oryzae* which infects rice plants, can grow normally on media supplemented with only pyruvate, a product of the gluconeogenic pathway but can only form virulent biofilms when supplemented with pentose or hexose sugars [35]. The role of biofilm formation in *C*Las is not well understood due to its fastidious nature, but it is likely to follow the *Xf* model.

### Sugar acids

Sugar acids were found commonly in our previous work with citrus phloem saps but in the nymph metabolome we found only traces of 2-ketogluconic acid, gluconic acid and saccharic acid. Each is distinguishable in silylated samples by its *m/z* 333 ion. Since the plant cell wall contains many of these sugar acids, they may be released in the phloem sap during phloem sap preparation [26].

### Sugar alcohols

Sugar alcohols, primarily *myo*-inositol and glucitol, were found in large amounts in nymph extracts, but there was no difference the levels between healthy and infected nymphs. On the contrary, two sugar alcohols found in smaller concentrations were significantly different. *Chiro*-inositol was up to 12-folds lower in infected nymphs (*p* = 0.016) while *scyllo*-inositol was 2-fold higher (*p*=.045). We found high levels of sugar alcohols, especially *myo*-inositol in *D*. *citri* haemolymph [20] and in citrus phloem saps [26]. In insects, sugar alcohols help in regulation of osmotic pressure [22,36]. They are acquired through feeding and are thought to act as osmoprotectants during cold acclimation and overwintering [37–39], and Although it is not known specifically if *C*Las utilizes *chiro*-inositol, the decrease found in infected nymphs suggests this possibility.

Inositol, a cyclic polyol, is easily phosphorylated and is the main carrier of phosphate in many living systems [37]. Phosphorylated inositol is incorporated in all eukaryotic cell membranes and some pathogenic fungi and bacteria as phosphatidylinositol (PI), or as phytic acid in plants [38]. Free inositol is synthesized from glucose-6-phosphate via myo-inositol-3-phosphate synthase [EC:5.5.1.4] followed by dephosphorylation by inositol monophosphatase [EC:3.1.3.25] [19,39,40].

Inositol can be synthesized *de novo* in the case of eukaryotes, or by cell membrane degradation by bacterial phytases [41]. These strategies are found in all *Actinobacteria*, but the *ino*1 gene which encodes myo-inositol-3-phosphate synthase seems to have been acquired by only a few other bacteria, perhaps by horizontal gene transfer from *Actinobacteria* [39,40]. In *Sinorhizobium meliloti*, a nitrogen fixing soil bacterium found in association with alfalfa and close relative of *C*Las [1], it was shown that a functional *myo*-inositol dehydrogenase gene was required for invasion of plant tissues and nodule formation [42]. In the same study, genes for *chiro*-inositol and *scyllo*-inositol dehydrogenases were identified for *S*. *melioti* and it was found that these inositol isomers could also be substituted as a sole carbon source.

There is also substantial evidence that inositol utilization is linked with pathogenicity factors in several bacterial genera including *Salmonella enterica* Typhimurium and *Escherichia coli* ED1a [40], *Mycobacterium tuberculosis* [39], and *Bacillus subtilis* [42]. In mycobacteria (*Actinobacteria*), sugar phosphates and PI are the structural components of the plasma membrane and cell wall and *M*. *tuberculosis* (the agent of TB) has at least one gene for dephosphorylating inositol; *M*. *smegmatis* has six homologs for inositol transporters [40]. Mutants of each of these were unable to utilize inositol or required high amounts of supplemental inositol to grow. Rothhardt et al [41]. found that four genes encoding inositol catabolism enzymes were induced only in the presence of inositol in *S*. *typhimurium* strain 14028, allowing degradation of the phospholipid membranes as a means of penetrating the cell membrane to further colonize the human gut. In addition, all pathogenic strains of *Cronobacter*, also a Gram-negative Proteobacteria sometimes found in contaminated food and infant formula, contained genes for inositol fermentation, while non-pathogenic strains did not [43]. The *C*Las genome annotation shows it has three inositol monophosphatase-related genes, but no *iol* dehydrogenases which would enable inositol utilization (https://www.ncbi.nlm.nih.gov/blast/txid537021). However, the *C*Las genome currently available is preliminary, and based on the bacteria found in one psyllid, *psy62*, not from a pure culture of *C*Las.

### Sugar phosphates

In the nymph metabolome, we detected three sugar phosphates, mostly in trace amounts. Only α-glycerophosphate (glycerol-3-phosphate) was reduced in response to *C*Las, perhaps as a result of increased glycolysis. I2P (inositol-2-phosphate, putative, *m/z* 217; 299; 319, RI 2382) increased slightly and *Myo*-inositol-5-phosphate (putative, *m/z* 211; 299; 315; 342, RI 2200) was increased significantly (*p* = 0.0007). In previous work, we detected erythrose-4-phosphate and G6P in adult *D*. *citri*, as well as phytic acid [14]. In the haemolymph we reported I2P and an unknown sugar phosphate (since confirmed as α-glycerophosphate) [20]. TMS-derivatized phosphates are easily distinguished in the chromatograms, as each has a distinctive *m/z* of 299 in the mass spectrum, the signature of the phosphate group. Free phosphate is usually detected in biological samples and reported as phosphoric acid (*m/z* 299; 314), which is relatively abundant compared to the small amounts of sugar phosphates reported. The roles of IP were discussed previously as key precursors for structural lipids.

### Fatty acids

Fatty acids (FAs) are critical for many biological functions in all living systems. Excess sugars and carbohydrates are converted to glycogen and lipids and stored in the adipocytes of the insect fat body [32]. Lipids are typically stored as triglycerides and are utilized for high energy demands such as diapause and extended flight [32], and are precursors for pheromones and eicosanoids, which are involved in innate immunity [44]. Fatty acids may make up half the mass of the fat body of some insects with 90% of the lipid component stored as triglyceride [32]. Indeed, in MCF-treated samples nymph samples, fatty acids represented >95% of the total ion chromatogram peak area, but < 1% of the total peak area for TMS-treated nymph extracts.

In *D*. *citri* nymphs we detected six different fatty acids as their methyl esters: palmitic acid (C16:0); palmitoleic acid (C16:1); stearic acid (C18:0); oleic acid (C18:1); linoleic acid (C18:2); and arachidic acid (C20:0). Levels of FA were elevated in response to *C*Las, especially stearic acid (2.18-fold, TMS), and 1.39-fold in MCF samples. Kruse et al. [45] reported that three lipases were downregulated in the gut of adult *D*. *citri* in response to *C*Las infection- hepatic triaglycerol lipase-like, phospholipase A-2 activating, and lysophospholipase-like protein 1, but these were not found to be differentially expressed in nymphs with or without *C*Las [5]. Five of the six FAs detected in infected nymphs by GC-MS increased (Table 2) compared to infected adults, suggesting a general upregulation of FA biosynthesis or lipase activity, but perhaps not enough to show a clear differential expression in the proteome analysis as found in Kruse et al. [45] If lipase activity is upregulated, we would expect to see a corresponding increase in glycerol, and, in fact, glycerol increased by 1.6 fold in response to *C*Las, but the difference was not significant (Table 1). Therefore, we predict that triglyceride lipase may be upregulated in *D*. *citri* nymphs in response to *C*Las. Additionally, storage levels of FAs in nymphs may be higher relative to adults due to demands for FAs during flight and reproduction that are not required during juvenile stages.

It should be noted that FA levels from MCF-derived samples showed levels 100–1000 times that of TMS-derivatized samples, owing to its better sensitivity toward FAs (Tables 1 & 2). The solvent extractions performed were intended to obtain the broadest range of all metabolites, i.e. not optimized for FA extraction. Using the data for MCF-treated samples, we found that oleic acid (9Z-Octadec-9-enoic acid) was the most abundant FA, while arachidic acid (eicosanoic acid, *m/z* 326) was least abundant. The unsaturated C18 FAs were not detected using TMS. All of the detected FAs except the C20:0 were also detected in citrus phloem saps, and C20:0 was not detected in adult *D*. *citri* haemolymph using MCF. It is not known if arachidic acid is nymph-specific or if we failed to detect it in the adult haemolymph due to the very small sample sizes available for study. This area of inquiry should be revisited with specific interest in the quantitative distribution of fatty acids in *D*. *citri* using optimized extraction and detection methods.

### Meta-analysis between *D*. *citri* adults and nymphs

**Fig 2:**
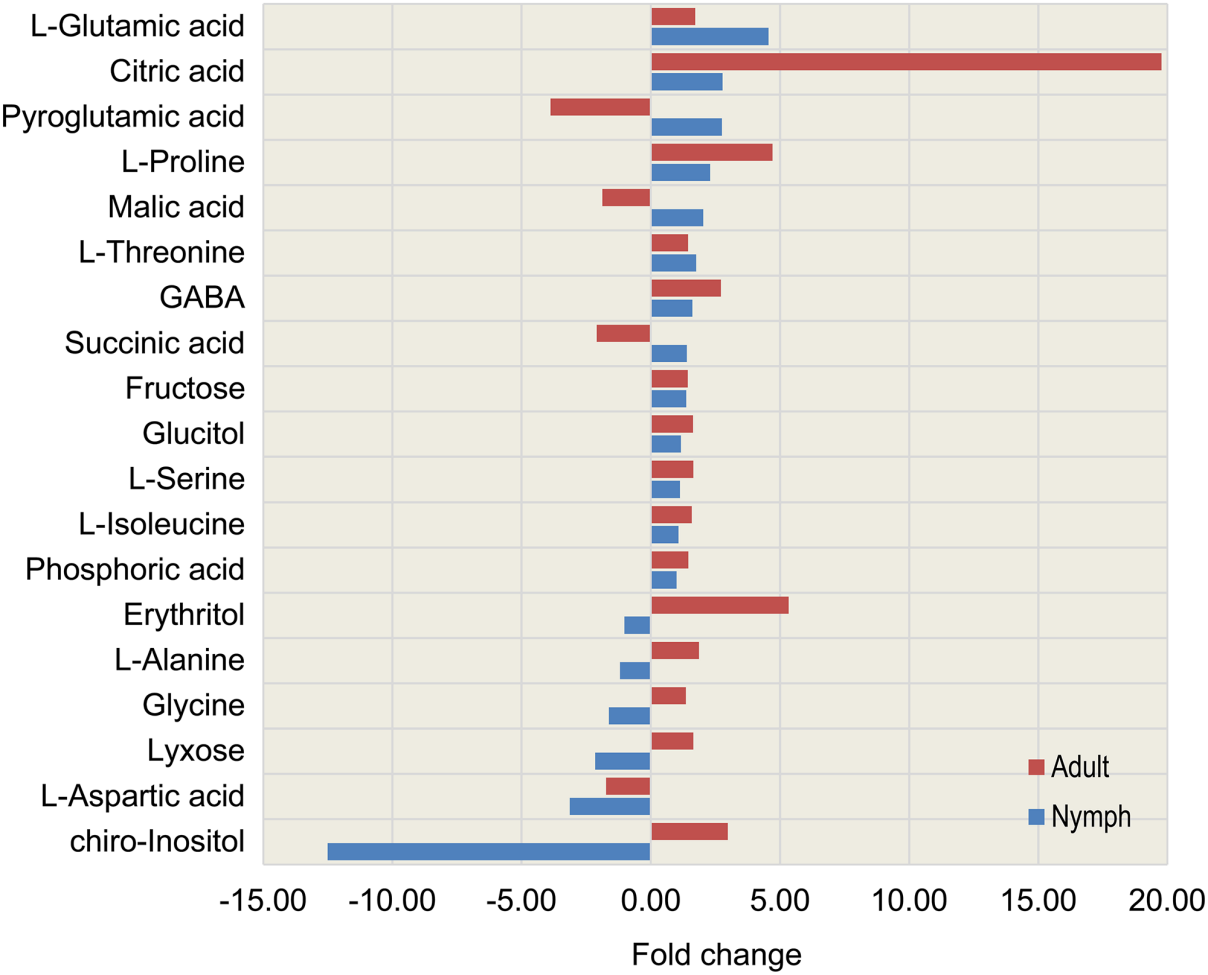
Fold changes of metabolite concentrations due to the effect of *C*Las infection on *D*. *citri* nymphs and adults. Data shown is for metabolites found to be in common (detected in both nymph and adult psyllid extracts by TMS derivatization) and significantly different between healthy and *C*Las-infected psyllid populations. Metabolomic data for adult psyllids was published previously [14].

When we compared the levels of metabolites which were found to be significantly different between *D*. *citri* nymphs and adults reared on healthy or *C*Las-infected Valencia trees, we found many similar trends. Fig 2 shows the fold changes of selected metabolites (those found in common among nymphal and adult psyllids). *C*Las infection increased the levels of 10 compounds in both nymphs and adults, while only one compound, L-aspartic acid, was decreased in both nymphs and adults. These effects must reflect specific metabolic demands placed on both nymphs and adults by *C*Las infection, either directly or indirectly. In adults, pyroglutamic acid, malic acid and succinic acid levels were lower than in nymphs. In nymphs, levels of L-alanine, glycine, erythritol, and lyxose were lower than in adults. *Chiro*-inositol decreased more than 12-fold in infected nymphs, suggesting utilization by the bacterium directly, or another direct affect due to *C*Las. Likewise, the large increase in citric acid in adult psyllids could be due to increased glycolysis rate induced by *C*Las as reported previously [14]. Lack of agreement in the direction of the fold changes for certain metabolites are most likely related to developmental differences between the nymph and adult stages, or due to specific metabolic demands placed on adults by activities such as flight and reproduction.

## Conclusion

In this study we report our findings on the metabolome of *D*. *citri* nymphs from methanolic extracts as analyzed GC-MS. Our goal was to be able to make recommendations from the details of this work for the development of an artificial media which would promote *C*Las growth *in vitro*. Holidic culture media must provide the necessary metabolites that would otherwise be induced *in vivo*. We found that nymphs reared on *C*Las-infected citrus were higher in many metabolites, especially those of the TCA cycle, C16 and C18 fatty acids, glucose, sucrose, and *myo*-inositol. It is difficult to elucidate whether the metabolites which changed in concentration were a result of a change in the feeding rate of nymphs, changes in the phloem sap quality of infected plants, accumulations of pathway intermediates due to altered metabolism, or due to direct utilization by *C*Las for growth within the nymph. Further study should be carried out to dissect these differences, and having the ability to culture the citrus greening bacterium would further elucidate these questions. Therefore, we recommend that nitrogen supplementation should include glutamic acid, pyroglutamic acid, phenylalanine, and serine. Acid components should include citric acid and/or malic acid, but there is some evidence that malate may inhibit growth. Formulations including sugar alcohols, especially *chiro*- and/or *scyllo*-inositol should be attempted along with *myo*-inositol, as we suspect direct utilization of these by *C*Las. Additionally, C16 and C18 fatty acids should be included, but C20 excluded for the reasons outlined.
